# Clinical characteristics and allergen detection of perioperative anaphylaxis: a 12-year retrospective analysis from an anesthesia clinic in China

**DOI:** 10.1186/s13741-021-00234-z

**Published:** 2022-01-21

**Authors:** Xiaowen Liu, Ruisong Gong, Xin Xin, Jing Zhao

**Affiliations:** 1 Department of Anesthesiology, China-Japan Friendship Hospital, Beijing, 100029 China; 2 Department of Anesthesiology, China-Japan Friendship Hospital, Chinese Academy of Medical Sciences & Peking Union Medical College, Beijing, 100029 China; 3 Department of Anesthesiology, Zhengding Union Hospital, Shijiazhuang, 050800 China

**Keywords:** Perioperative anaphylaxis, Diagnostic tests, Allergen, Alternative drugs, Repeat anesthesia

## Abstract

**Abstract:**

**Background:**

Anaphylaxis during anesthesia is a rare but often a potentially life-threatening event for patients. Identifying culprit agents responsible for anaphylaxis is of great important for avoiding potential re-exposure to allergens, but it poses great challenge for anesthetists. This retrospective study aimed to analyze the culprits of patients with a history of perioperative anaphylaxis referred to an anesthesia allergy clinic in China, and to evaluate the role of allergy diagnostic tests in clinical practice.

**Methods:**

A total of 145 patients (102 female/43 male) who attended the Anesthesia Allergy Clinic for allergen detection between 1 January 2009 and 31 December 2020 were reviewed retrospectively. Clinical characteristics, results of allergy diagnostic tests including skin, and/or basophil activation tests, and the incidence of repeat anaphylaxis after use of recommended alternative anesthetics were obtained.

**Results:**

Of these 145 patients, 109 patients (75.2%, 74 females/35 males) were determined to experience perioperative anaphylaxis. The most common presenting clinical feature was cardiovascular manifestations (*n* = 63, 57.8%). According to diagnostic work up, the most common causative agents for perioperative anaphylaxis were neuromuscular blocking agents (*n* = 35, 32.1%). After diagnostic work up, 52 patients underwent repeat anesthesia. None of these patients experienced recurrent anaphylaxis.

**Conclusions:**

This study suggests that neuromuscular blocking agents are the main cause of perioperative anaphylaxis. For patients with perioperative anaphylaxis, allergy diagnostic tests are essential to identify causative agents, and to find suitable alternative drugs for the future planning of subsequent anesthetics.

## Background

Perioperative anaphylaxis (POA) is a rare but often a potentially life-threatening condition that can contribute significantly to the morbidity and mortality of surgical patients (Au et al. 2020; Gonzalez-Estrada et al. 2021; Mertes et al. 2019). It is reported that the estimated worldwide incidence of POA varies from 1 in 1250 to 1 in 20,000 procedures, with a mortality rate of 4%, and an additional 2% surviving with severe brain damage (Gandhi et al. 2017; Mertes et al. 2019).

Identification of causative agents responsible for anaphylaxis is important to avoid potential re-exposure to allergens, but it poses a great challenge for anesthetists (Orihara et al. 2020). The evaluation of patients with POA must include a detailed history, comprehensive testing of all medications used in the perioperative period, and recommendation of alternative medications when a causative agent is identified (Carrion et al. 2020). Allergen testing includes the skin prick test (SPT), intradermal test (IDT), basophil activation test (BAT), specific IgE (sIgE), and others (Kalangara et al. 2021). Available literature from European and Australian populations suggests that neuromuscular blocking agents (NMBAs) are the most common cause of POA (Mertes et al. 2016). However, the relevance of those findings to Chinese patients has not been verified. Moreover, the effectiveness of diagnostic tests in identifying alternative agents for repeat anesthesia need to be further proved.

In China, a recent nationwide survey showed an incidence of perioperative anaphylaxis of one in 11,360, but allergen testing is rarely applied (Zhang et al. 2021). The Anesthesia Allergy Clinic at China-Japan Friendship Hospital is one of only a few specialized centers in China with advanced capabilities to perform allergen testing required to characterize POA. Thus, a retrospective analysis of anesthesia clinic referrals for POA across 2009 and 2020 was performed. Our aims were to describe the clinical characteristics of POA and results of diagnostic tests, and to review the incidence of repeat anaphylaxis for patients referred to our clinic after suspected POA and recommended alternative drugs.

## Methods

### Design and patient

This is a retrospective study on patients with a possible POA at an Anesthesia Allergy Clinic in Beijing, China, between 1 January 2009 and 31 December 2020. The study was approved by the Ethics Committee of China–Japan Friendship Hospital. As this is a retrospective study, informed consent of included patients was waived. The patients included in our study had to meet one of the following conditions: (1) experienced suspected POA or hypersensitivity or (2) had a history of uninvestigated reaction(s) associated with anesthesia.

First all patient histories were reviewed for likelihood for POA. At least two anesthesiologists in our team evaluated the patients for identification of POA by first excluding other possible causes such as hemorrhagic shock, asthma, or bronchospasm, and then assigning clinical scores for suspected perioperative immediate hypersensitivity reactions (Hopkins et al. 2019). POA was defined when there was at least one following criteria were met: (1) skin, mucosal tissue, or both (rash, erythema, pruritus or flushing, generalized hives, angioedema, swollen lips-tongue-uvula); (2) respiratory symptoms (dyspnea, wheeze, stridor, bronchospasm, chest tightness, hypoxemia, and reduced peak expiratory flow or increased ventilator pressures in intubated patients); (3) cardiovascular symptoms (tachycardia, cardiac arrhythmias, hypotension, shock or cardiac arrest). Patients with central nervous or digestive system symptoms that could not be explained by other reasons, such as dizziness, syncope or loss of consciousness, painful abdominal cramps or vomiting were also included (Atanaskovic-Markovic et al. 2019).

### Diagnostic work up

Following the guidelines of European Network on Drug Allergy, ENDA)/ (European Academy of Allergy and Clinical Immunology, EAACI) (Brockow et al. 2013; Mayorga et al. 2016), Six weeks to 6 months after the suspected POA, both in vitro (BAT) and/or in vivo (skin tests) tests were performed to identify potential causative agents (Brockow et al. 2002). All drugs (excepted inhalational anesthetics) adminstered during anesthesia were tested, alongside at least one corresponding alternative drug, to identify the causative agent and safe alternatives. Given the fact that few patients suspected of POA to antibiotics or latex attended our clinic, antibiotics and latex were not tested routinely. According to the medical history, the anaphylaxis did not happen when patients using antibiotics and latex, but it occured after the administration of anesthetics. A drug with a positive result of either skin tests or BAT was identified as the causative agent (Mertes et al. 2011).

### Skin tests

For consistency, test dilutions were prepared by two physicians. All dilutions were prepared and labelled in a sterile environment for individual patient use. Each skin prick was placed a minimum of 2 cm apart to reduce interference from adjacent positive tests. Normal saline was used as a negative control and morphine as a positive control. The concentrations of all drugs in the skin tests are shown in Table 1. All results were read at 15 to 20 min (Scolaro et al. 2017).

**Table 1:** Drugs concentrations for skin and basophil activation tests

Medications	Concentrations		
	Skin prick test (mg/ml)	Intradermal test (mcg/ml)	Basophil activation test(mcg/ml)
**Neuromuscular blocking agents**			
Cisatracurium	2	20	2.5
Rocuronium	10	100	500
Atracurium	2	20	2.5
Succinylcholine	10	100	2.5
**Opioids**			
Fentanyl	0.05	5	0.5
Sufentanil	0.005	0.5	0.5
Remifentanil	0.04	5	5
**Local anesthetics**			
Lidocaine	20	1 000	125
Ropivacaine	10	200	1 000
Bupivacaine	5	250	500
Articaine	40	1 000	1 000
**Sedatives**			
Midazolam	1	500	100
Propofol	10	1 000	500
Etomidate	2	200	200
Ketamine	50	5 000	1 000
**Others**			
Succinylated gelatin	1:1	1:10	1:10
Hydroxyethyl Starch	1:1	1:10	1:10
Dextran-40	1:1	1:10	1:100
Ondansetron	2	200	200
**Positive control**			
Morphine	1	100	N/A

The skin prick test (SPT) was performed on the forearm. Single allergen metal lancets were used to prick through a drop of allergen on the skin, deposit a drop of allergen into the skin. Enough pressure was required to cause a depression of 2 to 3 mm in the skin and hold for 1 s (Laguna et al. 2018). When the mean wheal diameter was larger than 3 mm and surrounded by erythema, the SPT was considered as positive; meanwhile, the saline control was negative (Bernstein et al. 2008).

The intradermal test (IDT) was performed with a 1.0 ml syringe attached to a 26 gauge hypodermic needle (Ebo et al. 2007). First, any air bubbles in the syringe were excluded before injection. The needle was directed at an angle of 5° to 10° to the skin surface. 0.02 to 0.05 ml of drug was injected intradermallly, raising a small bleb of 3 to 4 mm in diameter (Laguna et al. 2018). A pen was used to outline and the longest diameter of the bleb was measured. The result was considered as positive, if the wheal doubles in size or increases by 3 mm (Li et al. 2019b; Torres et al. 2001).

### Basophil activation test (BAT)

About 10 ml venous blood were obtained and placed into the K-EDTA tubes. Concentrations of all drugs used were listed in Table 1. Standard BAT protocol combined with reagent instructions were used, and Flow2 CAST kit (Bühlmann Laboratories AG, Schönenbuch, Switzerland) was employed. Both negative (stimulation buffer) and positive (anti-FcεRI mAb and formylmethionyl-leucyl-phenylalanine) controls were used for each sample. The results of BAT were expressed as net percentage upregulation in stimulated basophils compared with the negative control (% upregulation) (Eberlein et al. 2017). A cut-off of ≥ 5% activated basophils and a stimulation index ≥ 2 (SI = allergen stimulation divided by negative control) were considered to be positive results (Chen et al. 2016).

### Outcomes

Clinical characteristics including gender, age, allergy history, family history of allergy, type of anesthesia, and clinical manifestations were collected and assessed, as well as the results of diagnostic tests. Additionally, followed-up investigations were performed to obtain the incidence of recurrent perioperative anaphylaxis after diagnostic work up, and to evaluate the clinical benefits of allergen detection. If the second anesthesia can be safely performed based on the test results, it meaned that our test was correct and meaningful.

### Statistical analysis

IBM SPSS Statistics for Windows version 22.0 (IBM Corp, Armonk, NY, USA) and Microsoft Excel 2010 version were used for statistical analysis.

## Results

During the study period, 145 patients (102 female /43 male), age ranging from 1 to 78 years old, were referred to our clinic. 109 (75.2%) patients met our criteria for POA and 36 (24.8%) were excluded due to missing data or clinical presentation not consistent with POA.

The demographic characteristics of patients are shown in Table 2. Of 109 patients with confirmed POA, 69 (63.3%) had a history of allergy. Additionally, 18 patients reported a family history of allergy. The Clinical features of anaphylactic reaction of patients are summarized in Table 2. Among confirmed cases, the most common clinical presentations were cardiovascular manifestations (*n* = 63, 57.8%), including hypotension and cardiac arrhythmias, followed by respiratory (*n* = 52, 47.7%) and cutaneous symptoms (*n* = 37, 33.9%). To be specific, there were 43 patients (39.4%) who only presented one of three systemic symptoms, with 22 patients (20.2%), 5 patients (4.6%), and 16 patients (16.7%) only had cardiovascular, respiratory, and cutaneous symptoms, respectively. Besides that, 12 patients (11.0%) had both cardiovascular and respiratory symptoms, 16 patients (14.7%) had cardiovascular and cutaneous symptoms, and 13 patients (11.9%) had all of three systemic symptoms. A small number of patients (*n* = 18, 16.5%) had other clinical manifestations.

**Table 2:** Clinical characteristics of patients with confirmed POA

Characteristic	No. (%)
	Patients with a confirmed POA (***n*** = 109)
**Age (years)**^**a**^	43(1–78)
**Gender(*n*(%))**	
Male	35(32.1)
Female	74(67.9)
**Types of anesthesia**	
General anesthesia	69(63.3)
Non-general anesthesia	40(36.7)
**History of allergy**	
Only foods	10(9.2)
Only drugs	36(33.0)
Others	7(6.4)
Multiple	16(14.7)
None	40(36.7)
**Family history of allergy**	18(16.5)
**Clinical features**	
Cardiovascular	63(57.8)
Cutaneous	52(47.7)
Respiratory	30(27.5)
Other symptoms	18(16.5)

*POA* perioperative anaphylaxis

^a^Data presented as median (range)

Diagnostic tests were performed to identify the causative agents as well as suitable alternative drugs. Overall, of 109 patients with confirmed POA, 55 (50.5%) had identified culprits involving 89 agents. As shown in Table 3, SPT was performed in 13 patients, all of which were negative results; IDT was done for 90 patients, of which 51 patients (46.8%) were positive results; BAT were carried out in 103 patients, of which 17 patients (16.5 %) were positive.

**Table 3:** Laboratory tests of patients with possible and confirmed POA

Diagnostic tests	No. (%)	
	Total (***n*** = 145)	Patients with a confirmed POA (***n*** = 109)
**Skin prick test**		
Positive	0(0)	0(0)
Negative	16(11.0)	13(11.9)
Not done	129(89.0)	96(88.1)
**Intradermal test**		
Positive	59(40.7)	51(46.8)
Negative	66(45.5)	39(35.8)
Not done	20(13.8)	19(17.4)
**Basophil activation test**		
Positive	24(16.6)	17(15.6)
Negative	112(77.2)	86(78.9)
Not done	9(6.2)	6(5.5)

*POA* perioperative anaphylaxis

The diagnostic work up showed that NMBAs were the commonest causative agents for POA (*n* = 35, 32.1%), while sedatives ranked second (*n* = 25, 22.9%), followed by opioids (*n* = 15, 13.8%), local anesthetics (*n* = 10, 9.2%), and other agents (*n* = 4, 3.7%). Among the NMBAs involved, 23 were cisatracurium, 12 rocuronium, 7 atracurium, and 1 succinylcholine. Of sedatives involved, 20 were midazolam, 9 propofol, 2 etomidate, and 1 ketamine. Specially, the positive results of NMBAs and sedatives were mainly showed in IDT, which were different from opioids and local anesthetics. Table 4 described all the drugs involved. In addition, alternative drugs were recommended for all 55 patients who had confirmed culprits.

**Table 4:** Drugs tested to be responsible for POA

Tested drug	Positive results (no.)		
	Intradermal test	Basophil activation test	both
**Neuromuscular blocking agents**	**33**	**5**	**3**
Cisatracurium	23	1	1
Rocuronium	7	4	2
Atracurium	2	0	0
Succinylcholine	1	0	0
**Opioids**	**11**	**5**	**1**
Fentanyl	5	3	0
Sufentanil	5	1	1
Remifentanil	1	1	0
**Local anesthetics**	**5**	**6**	**1**
Lidocaine	4	4	1
Ropivacaine	0	1	0
Bupivacaine	1	0	0
Articaine	0	1	0
**Sedatives**	**18**	**9**	**2**
Midazolam	13	4	1
Propofol	3	3	0
Etomidate	1	2	1
Ketamine	1	0	0
**Others**	**4**	**3**	**3**
Succinylated gelatin	3	3	3
Ondansetron	1	0	0

*POA* perioperative anaphylaxis

Up to 31 December 2020, 80 (73.4%) out of the 109 patients with a confirmed POA were followed up to observe whether recurrent anaphylaxis occur. Of them, 46 experienced repeat anesthesia, including 19 who had identified culprits and were suggested alternative drugs. The repeat anesthesia of all patients proceeded uneventfully, with no further anaphylaxis or complications.

## Discussion

There is fairly limited data about epidemiology and clinical management of POA in China. From 2009 to 2020, our research team used both skin tests and BAT for assessment of perioperative allergens to identified causative agents in 55 cases out of 109 patients diagnosed with POA. The results demonstrated that these diagnostic tests were useful methods to find allergens responsible for POA. To our best knowledge, there are only a few anesthesia allergy research teams with ability to perform both in vivo and in vitro diagnostic tests, and this is the first retrospective study describing data on perioperative allergen detection in mainland of China.

Recent studies showed that the incidence of POA is higher than thought (Au et al. 2020). As the included patients were from different hospitals, this study was not able to provide the incidence of POA in China. However, some significant demographic features were still provided. For example, this study indicates that females are more likely to experience POA, which is consistent with other reported studies (Harboe et al. 2005; Huang et al. 2019). For example, a Singaporean study indicated that the female-to-male percentage for POA was 56.3:43.8 (Harboe et al. 2005). Similarly, a 3:1 female-to-male ratio was also reported in a Norwegian study (Huang et al. 2019). A higher incidence of POA in females may be due to cross-reactivity with cosmetics, which females are more commonly exposed to than males (Harboe et al. 2005). Cosmetics contain the quaternary ammonium group, which is an important structural component of available NMBAs. In addition, 63.3% of patients with a confirmed POA had a history of allergy, which is much higher than the data reported in the above Singaporean study (Harboe et al. 2005). It has been confirmed that a history of drug, food and other substances is a significant risk of POA (Ebo et al. 2019). Thus, allergy evaluation followed by anti-allergy premedication may play an important role in prevention of POA.

As POA is a consequence of multiple potential pathophysiological mechanisms and has heterogeneous clinical presentations (Harper et al. 2018), clinical manifestations and intraoperative diagnostic tests such as serum tryptase are needed to confirm the occurrence of POA. However, the serum tryptase test is rarely applied in China. As such, only clinical manifestations serve as the clue for diagnosis of POA in most cases and this poses a great challenge for subsequent clinical management. Consistent with previous reports (Patil et al. 2020), this study showed that the most common clinical manifestations of patients with POA were cardiovascular symptoms, accounting for 57.8%, followed by respiratory and cutaneous symptoms. The immediate-phase response's proinflammatory mediators such as histamine, neutrophil and eosinophil chemotaxis factors, and proteolytic enzymes, are responsible for many clinical symptoms (Justiz et al. 2020). It is generally believed that the mediators promote histological changes. For example, histamine and lipid mediators can cause vascular leakage, and subsequent hypovolemia leading to reduced venous return and circulatory failure (Harper et al. 2018; Haybarger et al. 2016).

Avoidance of re-exposure to triggers of POA is critical to safety for subsequent anesthesia. However, it is difficult to find the culprits because many anesthetics are administered simultaneously during anesthesia. Diagnostic tests, such as skin tests, BAT, and specific IgE, are the most crucial methods of allergen detection. It is recommended that skin tests including SPT and IDT should be applied to all cases with a clinical history supporting diagnosis of POA (Laguna et al. 2018). Despite a high positive predictive value of diagnostic tests in the patient with clinical findings consistent with POA (Takazawa et al. 2019), the present study confirms that SPT are often negative.

BAT is in vitro diagnostic procedure, which has been suggested as useful supplements to skin tests, as it has the advantage with no risk of not triggering immediate hypersensitivity reactions (Mertes et al. 2011). It is reported that BAT has a high diagnostic accuracy in identifying culprit agents of POA, with sensitivities of 50–90% and specificities > 90% (Ebo et al. 2018).

Our data showed that the results of skin tests and BAT significantly differed in various drugs, especially for the NMBAs and sedatives. Indeed, previous work revealed that sensitization of skin tests and BAT completely matched only in 15% of patients (Li et al. 2019a). Kim’s study showed the BAT yielded positive results in 57.9% of the cases, which was similar to the results of SPT and IDT (42.1% and 57.9%, respectively) (Kim et al. 2016). It is worth noting that the positive rates of IDT and BAT in our study were 46.8% and 16.5%, respectively, which are significantly lower than previously published data. The main reasons for low diagnostic sensitivity of these tests are that they are mainly used for IgE-mediated allergy, and a considerable number of cases in this study might be non-IgE-mediated allergy. Another possible explanation for these different results might be not all materials were tested in our diagnostic work up. To improve the efficacy of identifying culprits (Mertes et al. 2011), an integration of both BAT and skin tests was adopted for evaluation of POA in this study.

Our results showed that NMBAs were the main causative agents of POA, which is consistent with the findings of previous studies from other countries (Di Leo et al. 2018; Eberlein et al. 2017; Petitpain et al. 2018). Hypersensitive reaction to NMBAs may be either IgE or not-IgE-mediated. The IgE-mediated hypersensitive response is mainly attributable to the quaternary ammonium structures that represent the main antigenic epitope of NMBAs (Di Leo et al. 2018). Environmental chemicals, such as cosmetics with quaternary ammonium structures, are responsible for anaphylactoid reaction(Rouzaire et al. 2013). In the general population, even in the absence of clinical signs or symptoms, 9.3% of patients tested have a positive skin test for specific IgE quaternary ammonium ions as in NMBAs (Hepner and Castells, 2003). Among the four NMBAs tested in our clinic, cisatracurium represented the first cause of POA, followed by rocuronium, atracurium, and succinylcholine. Recent work indicates isolated instances of modestly increased histamine levels after cisatracurium administration, which may cause POA (Berroa et al. 2015). Cisatracurium can also trigger mast cell degranulation and the release of pro-inflammatory mediators through MRGPRX2 (Che et al. 2018). In addition, cisatracurium can cause systemic allergic reactions (Au et al. 2020). Besides, epitopes that are ubiquitous in NMBAs (such as substituted ammonium groups) lead to high cross-reactivity between these drugs (most consistently between pancuronium, rocuronium and vecuronium) (Di Leo et al. 2018). Previous exposure to non-anesthetic drugs may cause covert sensitization to NMBAs, resulting in reactions among patients without prior anesthesia.

It should be realized that the fundamental goal of allergen detection is avoidance of reexposure to culprits, and to ensure safety of subsequent anesthesia and surgery without allergic risk. Our study demonstrated that repeat anesthesia was safely performed in all patients receiving subsequent surgery, with none experiencing recurrent POA. This suggests that skin tests combined with BAT are useful for finding causative agents and suitable alternative drugs. However, further studies are still needed to prove our findings, due to insufficient sample size and data quality.

Overall, our study emphasizes the importance of referral procedure, accompanied by anesthetic information and allergy tests in identifying potential culprits, which is a effective way to prevent the recurrence of perioperative anaphylaxis. If anaphylaxis is suspected, intravenous epinephrine administration and fluid therapy are main treatments. Then, glucocorticoid and antihistamines can also be used to help reduce symptoms (Manian and Volcheck, 2021). Although there is much diagnostic uncertainty, analysis of data on the outcome of repeat anesthesia and its congruence with results published by others validates our approach to the investigation of POA. For patients attending our clinic, it enables us to quantify future risk of POA after an assessment in our clinic and provides a benchmark for other anesthesiologists.

## Limitations

There are some inherent limitations due to retrospective nature of our study. First, severity of POA was not quantified, and the treatment measures were not been completely recorded. Second, as some patients were lost follow-up at the time of publication, not all patients completed the entire work-up investigations. Third, limited by conditions, only skin tests and BAT were used in this study, other diagnostic tests, such as tryptase and sIgE, were not applied. It is generally considered that measurement of serum tryptase is useful for establishing a differential diagnosis (Beck et al. 2019). Furthermore, sIgE can be carried out easily, if determination kits are available (Mertes et al. 2011). Thus, we recommend that both tryptase and sIgE should be integrated diagnostic tests for identification of POA culprits and alternative drugs, if the conditions allow.

## Conclusions

This retrospective analysis from mainland of China demonstrates that females make up the majority of the POA crowd. The most common clinical manifestations of patients with a POA are cardiovascular symptoms and NMBAs are identified as main culprit agents for POA. An integration of skin tests and BAT into allergen detection can enable anesthetists to find safe alternative anesthetics for subsequent surgery.

## Data Availability

The datasets used and/or analyzed during the current study are available from the corresponding author on reasonable request.

## Abbreviations

POA: Perioperative anaphylaxis
SPT: Skin prick test
IDT: Intradermal test
BAT: Basophil activation test
sIgE: Specific IgE
NMBAs: Neuromuscular blocking agents
ENDA: European network on drug allergy
EAACI: European academy of allergy and clinical immunology
